# Linked dimers of the AAA+ ATPase Msp1 reveal energetic demands and mechanistic plasticity for substrate extraction from lipid bilayers

**DOI:** 10.1002/1873-3468.70187

**Published:** 2025-10-13

**Authors:** Deepika Gaur, Brian Acquaviva, Baylee A. Smith, Nathan Walker, Isabella Walter, Matthew L. Wohlever

**Affiliations:** ^1^ Department of Cell Biology University of Pittsburgh PA USA; ^2^ Department of Chemistry and Biochemistry University of Toledo OH USA; ^3^ Department of Microbiology University of Illinois Champaign IL USA; ^4^ Department of Molecular Genetics Ohio State University Columbus OH USA

**Keywords:** ATPase associated with diverse cellular activities (AAA+), lipid bilayer, membrane protein, mitochondria, proteostasis

## Abstract

Msp1 is a membrane‐anchored AAA+ (ATPases Associated with diverse cellular Activities) enzyme that extracts membrane proteins from lipid bilayers. To understand how the subunits in the homohexamer convert ATP hydrolysis into mechanical work, we developed covalently linked dimers combining wild‐type and catalytically inactive (E193Q) subunits. These assembled into pseudohexameric trimers of dimers and retained ATPase activity, indicating that E193Q does not act as a dominant negative for ATP hydrolysis. Conversely, substrate extraction was impaired in some constructs, suggesting position‐specific effects. Surprisingly, constructs with a twofold difference in ATPase rates showed minimal differences in substrate extraction across lipid environments, suggesting excess ATPase capacity. These findings clarify how Msp1 coordinates hydrolysis, its energetic requirements, and substrate access to the pore.

## Graphical abstract

Cells must clear mislocalized or faulty proteins from membranes to survive. The AAA+ ATPase Msp1 performs this task, but dissecting how its six subunits work together is challenging. We engineered linked dimers with varied numbers of functional subunits to reveal how Msp1 subunits cooperate and use energy to extract proteins from the lipid bilayer.

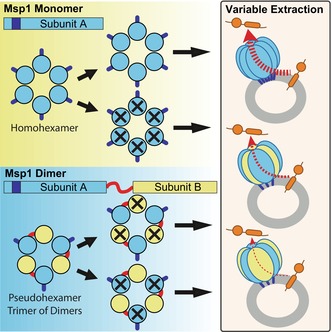

## Abbreviations

**AAA+**, ATPase associated with diverse cellular Activities

**ADP**, adenosine diphosphate

**ATP**, adenosine triphosphate

**EQ**, E193Q walker B mutation (ATP hydrolysis deficient)

**ER**, endoplasmic reticulum

**His**_**6**_ **/His**_**10**_, polyhistidine affinity tags

**OMM**, outer mitochondrial membrane

**SC/2R**, sequential clockwise/2‐residue step

**SEC**, size exclusion chromatography

**TMD**, transmembrane domain

**TMH**, transmembrane helix

**WT**, wild‐type

Many fundamental cellular processes such as DNA replication, protein degradation, and vesicle trafficking require the physical remodeling of macromolecules. This energy‐intensive process is carried out by the ATPases Associated with diverse cellular Activities (AAA+) family of molecular motors, which use the free energy of ATP binding and hydrolysis to perform mechanical work [1, 2, 3, 4, 5, 6]. Classical AAA+ proteins are typically hexamers and undergo ATP‐dependent movements to translocate a substrate through a narrow axial pore, resulting in substrate unfolding [7, 8, 9, 10, 11, 12].

The relationship between ATP hydrolysis and substrate processing intimately depends on the coordination of ATP hydrolysis between subunits in the AAA+ hexamer. Cryo‐EM structures of diverse AAA+ proteins typically show a right‐handed spiral arrangement of subunits that form a lock washer [13, 14]. While the exact details vary depending on the AAA+ protein and particular combination of ATP analog and ATPase‐deficient mutants used, these structures generally have five ATP/ADP bound subunits arranged in a spiral staircase and a transitional sixth subunit in the apo state [9, 15, 16]. The substrate is gripped in the central pore by highly conserved loops, referred to as pore loops, which intercalate between the side chains of the substrate [17]. The pore loops are arranged like a spiral staircase with a two‐amino‐acid step size [13]. These structures suggest that ATP hydrolysis occurs in an ordered sequence around the ring [18]. As subunits proceed through the steps of ATP binding, hydrolysis, and release, they transition up the spiral, thereby translocating the substrate. This mechanism has been termed the sequential clockwise/2‐residue step (SC/2R) mechanism [8, 19].

While there is strong structural support for the SC/2R mechanism, biochemical experiments suggest that many AAA+ proteins may not operate strictly through the SC/2R mechanism. For example, because the SC/2R mechanism requires a sequential ordering of ATP hydrolysis, this model predicts that a single inactive subunit will likely act as a dominant negative. However, an engineered version of the AAA+ protein ClpX containing multiple inactive subunits is capable of translocating substrates [20]. This raises the possibility that the coordination of ATP hydrolysis may depend on the specific AAA+ protein and/or the type of substrate being processed. Extraction of membrane proteins from the lipid bilayer is one essential but underexplored area of AAA+ protein function [21]; thus, we sought to understand the mechanism and coordination of ATP hydrolysis by a AAA+ membrane extractase.

Msp1 is homohexameric AAA+ ATPase anchored on the outer mitochondrial membrane (OMM) and peroxisomes that promotes protein quality control by processively extracting mislocalized proteins or substrates that have become stalled in the Translocase of the Outer Membrane (TOM) complex [22, 23, 24, 25, 26, 27, 28, 29]. The extracted substrates are then transferred to the ER where they are mostly ubiquitinated and degraded [30, 31]. Loss of Msp1 results in severe mitochondrial stress including accumulation of mislocalized proteins, mitochondrial fragmentation, and loss of oxidative phosphorylation [32, 33]. The human homolog ATAD1 has also been shown to disassemble the AMPA Receptor and regulate apoptosis by extracting the pro‐apoptotic protein BIM [34, 35].

Because there are robust *in vitro* and cellular assays for Msp1/ATAD1 activity [15, 32, 36], we sought to use Msp1 as a model system to understand the mechanism of transmembrane helix (TMH) extraction by AAA+ proteins. In addition to understanding the coordination of ATP hydrolysis between subunits, another important topic is understanding how the rate of ATP hydrolysis affects substrate processing. Previous studies on other AAA+ unfoldases revealed that mechanical substrate unfolding is governed by a kinetic competition between substrate translocation and refolding of partially unfolded intermediates, with robust substrate unfolding requiring several rapid rounds of ATP hydrolysis [37, 38, 39, 40, 41]. It is unclear whether a similar kinetic competition between TMH extraction and re‐insertion also governs Msp1 activity.

**Fig. 1:**
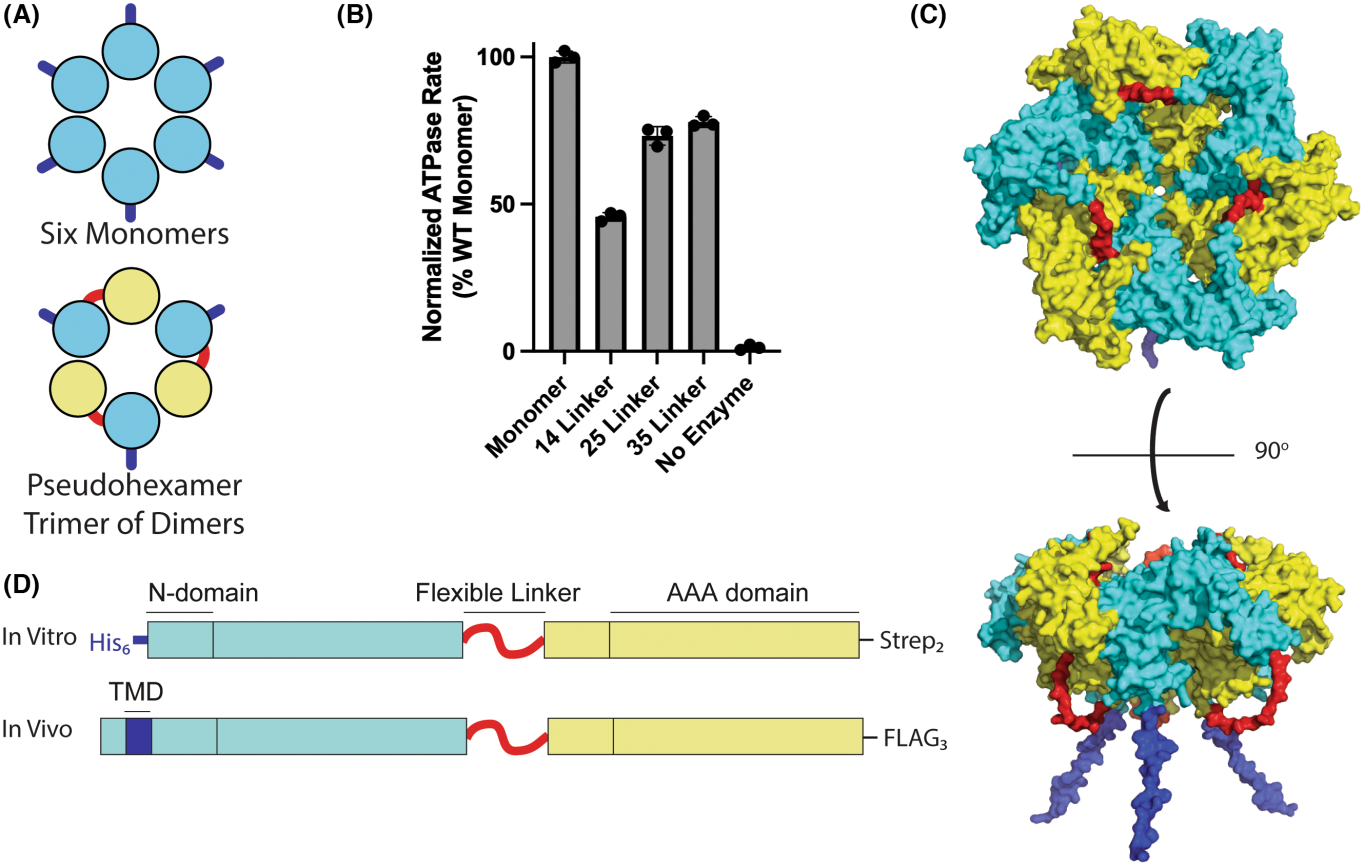
Design of covalently linked dimers. (A) Diagram showing how covalently linked dimers allow for the design of subunit specific mutations by forming a trimer of dimers. The membrane anchor is dark blue, subunit 1 is cyan, the covalent linker is red, and subunit 2 is yellow. (B) ATPase assay shows that dimers with a linker length of 35 residues are most active. ATPase rates were measured with 2 mm ATP and normalized to that of the WT monomer. None of the constructs in this figure had C‐terminal Strep tags. Error bars are standard deviation from three replicates. (C) The top AlphaFold3 model of the WT‐WT hexamer shows the expected arrangements of subunits. Coloring of subunits and features is the same as A. (D) Diagram showing the design of the covalently linked dimers for use *in vitro* and *in vivo*. Coloring of subunits and features is the same as A.

A major obstacle for studying the coordination of ATP hydrolysis in Msp1 homohexamers is the inability to generate subunit‐specific mutations. Drawing inspiration from previous work on the AAA+ ATPase ClpX [20], we sought to overcome this challenge by using genetically encoded flexible linkers to create covalent Msp1 dimers (Fig. 1A). The resulting constructs form a pseudohexameric trimer of dimers rather than a standard hexamer composed of six monomers, thereby allowing the generation of subunit‐specific mutations.

Here, we developed covalently linked Msp1 dimers containing varying combinations of wild‐type (WT) and ATPase‐deficient Walker B mutant (E193Q) subunits. These constructs have different maximum rates of ATP hydrolysis, which allow us to probe the ATP hydrolysis requirements for Msp1 functionality both *in vitro* and *in vivo*. Our results demonstrate that hexamers with up to 50% inactive subunits retain robust activity, suggesting that Msp1 has mechanistic plasticity to bypass inactive subunits. Overall, our results provide foundational insights into how Msp1 coordinates ATP hydrolysis between six subunits to overcome the substantial thermodynamic barriers that accompany substrate extraction from a lipid bilayer.

## Methods

### Msp1 construct design and cloning

Soluble Msp1 dimers had a deletion of the first 32 residues to remove the TMD [26] and also contained a noncleavable N‐terminal His_6_ and a C‐terminal twin‐Strep. Dimers were cloned into a Golden Gate domesticated pET28b vector containing an N‐terminal His_10_ affinity tag followed by bdNedd8 solubility tag [42]. The construct design allows for multiple tags for tandem affinity purification, removal of the solubility tag via cleavage with bdNEDP1 protease [43], and a noncleavable His_6_ tag that is used to anchor soluble Msp1 to liposomes for the *in vitro* extraction assay. Dimers were generated by gene synthesis such that each subunit has the same protein sequence but a unique DNA sequence. The linker sequence is ASGSGGSEASASAGAAGSGDGSGSGGSEGGTSGAT and contains a HindIII restriction cloning site. To generate point mutations, the individual subunits can be subcloned into a pET28b vector, mutated by Quikchange PCR, and then restriction cloned back into the dimer vector.

### Protein expression and purification

#### Protein expression

Briefly, constructs were transformed in BL21 pRIL cells, grown in terrific broth at 37 °C until OD_600_ = ~0.6–0.8. Cells were induced with 250 μm isopropyl β‐D‐1‐thiogalactopyranoside (IPTG) overnight at 18 °C for the Sec22 substrate or 16 °C for all other constructs. Cells were pelleted by centrifugation, resuspended in appropriate Lysis Buffer supplemented with 0.05 mg·mL^−1^ Lysozyme and 1 mm phenylmethylsulfonyl fluoride (PMSF), and stored at −80 °C. Msp1 pellets were also supplemented with 1 mm Protease Inhibitor (Pierce) prior to storage at −80 °C. For purification, the cell pellet was rapidly thawed, supplemented with 500 U of universal nuclease (Pierce), lysed by sonication, and the supernatant was isolated by centrifugation for 30 min at 18 000 ***g***.

#### SGTA and calmodulin

Lysates for GST‐SGTA and GST‐calmodulin were loaded onto a gravity column containing glutathione resin (Thermo Fisher, Waltham, MA, USA). Resin was washed with 15 CV of SGTA Lysis Buffer (50 mm Hepes‐KOH pH 7.5, 150 mm NaCl, 1 mm DTT, 0.01 mm EDTA, 10% glycerol). Protein was eluted with 3 CV of SGTA Lysis Buffer supplemented with 10 mm reduced glutathione. The protein was further purified by size exclusion chromatography with a Superdex200 Increase 10/300 GL column equilibrated in SGTA FPLC Buffer (20 mm Hepes‐KOH pH 7.5, 100 mm NaCl, 0.1 mm TCEP). Pure fractions, as judged by SDS/PAGE, were pooled, spin‐concentrated to 15–30 mg·mL^−1^ in an Amicon Ultra centrifugal filter (Pierce), aliquoted, and flash frozen in single‐use aliquots. Protein concentration was measured by absorbance at 280 nm using the extinction coefficient from Expasy Protparam [44].

#### LgBiT and MBP‐ubiquitin‐mNG2

LgBiT and MBP‐Ubiquitin‐mNG2 were purified by Ni‐NTA affinity chromatography. Cleared lysate was loaded onto a gravity column containing Ni‐NTA resin (Qiagen, Hilden, Germany) and washed with 15 CV of LgBiT Lysis Buffer (20 mm Tris pH 7.5, 200 mm NaCl, 20 mm imidazole, 1 mm DTT, 0.01 mm EDTA) followed by 5 CV of LgBiT Wash Buffer (20 mm Tris pH 7.5, 200 mm NaCl, 50 mm imidazole, 1 mm DTT, 0.01 mm EDTA). Samples were eluted with LgBiT Elution Buffer (20 mm Tris pH 7.5, 200 mm NaCl, 250 mm imidazole, 1 mm DTT, 0.01 mm EDTA) and spin‐concentrated to 5–10 mg·mL^−1^ in an Amicon Ultra centrifugal filter (Pierce). LgBiT was cleaved with a 0.01 mg·mg^−1^ ratio of 3C protease to substrate overnight at 4 °C. Samples were further purified by size exclusion chromatography with a Superdex200 Increase 10/300 GL column equilibrated in LgBiT FPLC Buffer (20 mm Tris pH 7.5, 200 mm NaCl, 1 mm DTT). Pure fractions, as judged by SDS/PAGE, were pooled, spin‐concentrated to 10–15 mg·mL^−1^ in an Amicon Ultra centrifugal filter (Pierce), aliquoted, and flash‐frozen in single‐use aliquots. Protein concentration was measured by absorbance at 280 nm using the extinction coefficient from Expasy Protparam [44].

#### Msp1 constructs

Msp1 constructs were expressed, harvested, and lysed as described above; except, following lysis, buffer was supplemented with 500 mm sodium chloride and 1% n‐dodecyl‐β‐d‐maltoside and incubated at 4 °C for 5 h prior to centrifugation at 18 000 ***g*** [26]. Clarified supernatant was loaded onto a gravity column with Ni‐NTA resin (Qiagen) and washed with 20 column volumes (CV) of Msp1 Lysis Buffer (20 mm Tris pH 7.5, 200 mm potassium acetate, 20 mm imidazole, 1 mm DTT, 0.01 mm EDTA) followed by 10 CV of Msp1 Wash Buffer (20 mm Tris pH 7.5, 100 mm potassium acetate, 30 mm imidazole, 1 mm DTT, 0.01 mm EDTA). Samples were eluted with 3 CV of Msp1 Elution Buffer (20 mm Tris pH 7.5, 200 mm potassium acetate, 250 mm imidazole, 1 mm DTT, 0.01 mm EDTA).

Protein concentration was estimated by absorbance at 280 nm using the extinction coefficient from ExPASy Protparam [45] and incubated at 4° for 2–6 h with 50:1 (w/w) bdNEDP1 protease. Protein was loaded onto a gravity column with Strep‐Tactin resin (IBA) and washed with 20 CV of Msp1 Dialysis Buffer (20 mm Tris pH 7.5, 200 mm potassium acetate, 1 mm DTT). Samples were eluted with 2.5 CV of Strep Elution Buffer (100 mm Tris pH 8.0, 150 mm NaCl, 1 mm EDTA, 50 mm biotin, 1 mm DTT) and dialyzed against 1:100 (v/v) Msp1 Dialysis Buffer at 4°, either once overnight or twice for 2 h each. Samples were spin‐concentrated to ~2 mg·mL^−1^ in a Thermo spin concentrator (Pierce), aliquoted, and flash‐frozen in single‐use aliquots. Protein concentration was measured by absorbance at 280 nm using the extinction coefficient from ExPASy Protparam [45].

#### SumoTMD substrate

The SumoTMD substrate was expressed, harvested, and lysed as described above using the SumoTMD Lysis Buffer (50 mm Tris pH 7.5, 300 mm NaCl, 10 mm MgCl_2_, 10 mm imidazole, 10% glycerol). After sonication, membrane proteins were solubilized by the addition of n‐dodecyl‐β‐d‐maltoside (DDM) to a final concentration of 1% and rocked at 4 °C for 30 min. Lysate was cleared by centrifugation at 35 000 ***g*** for 1 h. Cleared lysate was loaded onto a gravity column with Ni‐NTA resin (Qiagen) and washed with 5 CV of SumoTMD Wash Buffer 1 (50 mm Tris pH 7.4, 500 mm NaCl, 10 mm MgCl_2_, 10 mm imidazole, 5 mm β‐mercaptoethanol (BME), 10% glycerol, 0.1% DDM). Resin was washed with 5 CV of SumoTMD Wash Buffer 2 (50 mm Tris pH 7.4, 300 mm NaCl, 10 mm MgCl_2_, 25 mm imidazole, 5 mm BME, 10% glycerol, 0.1% DDM) and 5 CV of SumoTMD Wash Buffer 3 (50 mm Tris pH 7.4, 150 mm NaCl, 10 mm MgCl_2_, 50 mm imidazole, 5 mm BME, 10% glycerol, 0.1% DDM). Sample was then eluted with 2 CV of SumoTMD Elution Buffer (50 mm Tris pH 7.5, 150 mm NaCl, 10 mm MgCl_2_, 250 mm imidazole, 5 mm BME, 10% glycerol, 0.1% DDM). Samples were spin‐concentrated in an Amicon Ultra centrifugal filter (Pierce) and cleaved with a 0.01 mg·mg^−1^ ratio of 3C protease/substrate overnight.

On day 2, the sample volume was brought up to 5 mL using SumoTMD FPLC Buffer (50 mm Tris pH 7.4, 150 mm NaCl, 10 mm MgCl_2_, 5 mm BME, 10% glycerol, 0.1% DDM). To remove uncleaved protein and 3C protease, samples were incubated with Ni‐NTA resin for 30 min and loaded onto a column. The flow through was collected and spin‐concentrated. Samples were further purified by size exclusion chromatography with a Superdex200 Increase 10/300 GL column equilibrated in SumoTMD FPLC Buffer. Pure fractions, as judged by SDS/PAGE, were pooled, spin‐concentrated to 5 mg·mL^−1^ in an Amicon Ultra centrifugal filter (Pierce), aliquoted, and flash‐frozen in single‐use aliquots. Protein concentration was measured by absorbance at 280 nm using the extinction coefficient from Expasy Protparam [44].

### ATPase assays

ATPase activity was determined using a coupled ATPase assay modified from previous work on ATPases [46]. Reactions were set up in a 96‐well clear bottom plate. Each reaction contained a final concentration of 50 mm Hepes‐KOH (pH 7.5), 200 mm potassium acetate, 2 mm DTT, 1 mm Phosphoenolpyruvate, 0.3 mm NADH, 20 U·mL^−1^ Lactate Dehydrogenase, 10 U·mL^−1^ Pyruvate Kinase, 1 μm of the desired Msp1 construct normalized to hexamer concentration and between 0 and 2 mm ATP. Reactions were performed in triplicate. The plate was incubated at 30 °C for 15 min. The reaction was initiated by the addition of 100 mm magnesium acetate to a final concentration of 10 mm. Absorbance at 340 nm was read every 10 s for a 10‐min period. Kinetic parameters were determined by fitting data to the Hill form of the Michaelis–Menten equation *V* = *V*_MAX_/(1 + (*K*_M_/[S])^h^).

### Liposome preparation and reconstitution

Liposomes were prepared as described previously [36]. Briefly, a lipid film was prepared by mixing the indicated chloroform stocks of lipids with 1 mg of DTT. The 18:1 liposomes contained synthetic 18:1, 18:1, trans phosphatidyl choline (Avanti 850376C) and synthetic 18:1, 18:1 1,2‐dioleoyl‐sn‐glycero‐3‐[N‐((5‐amino‐1‐carboxypentyl)iminodiacetic acid)succinyl] Nickel salt (Avanti 790 404) at a 98:2 molar ratio. The 18:2 liposomes contained synthetic 18:2, 18:2 phosphatidyl choline (Avanti 850385C) and synthetic 18:1, 18:1 1,2‐dioleoyl‐sn‐glycero‐3‐[N‐((5‐amino‐1‐carboxypentyl)iminodiacetic acid)succinyl] Nickel salt (Avanti 790 404) at a 98:2 molar ratio.

Chloroform was evaporated under a gentle stream of nitrogen and then left on a vacuum (<1 mTorr) overnight. Lipid film was fully resuspended in Liposomes Buffer (50 mm Hepes KOH pH 7.5, 15% glycerol, 1 mm DTT) over the course of several hours by vortexing and rotation on a wheel at room temperature. The final concentration of liposomes was 20 mg·mL^−1^. The liposomes were subjected to five freeze–thaw cycles with liquid nitrogen and rapid thawing followed by 15× extrusion through a 100 nm filter at 60 °C. Single‐use aliquots were flash frozen in liquid nitrogen and stored at −80 °C.

SumoTMD construct was reconstituted into liposomes by mixing 2.5 mm SumoTMD and 2 mg·mL^−1^ liposomes. The final volume was brought to 100 mL with Reconstitution Buffer (50 mm Hepes‐KOH pH 7.5, 200 mm potassium acetate, 7 mm magnesium acetate, 2 mm DTT, 10% sucrose, 0.01% sodium azide, and DeoxyBigChap (DBC)). The concentration of DBC used for reconstitution was re‐optimized for each liposome preparation but ranged from 0.1%–0.5%. Detergent was removed by adding 25 mg of biobeads and rotating the samples for 16 h at 4 °C. After removing the biobeads, the reconstituted material was incubated with 5 mm GST‐SGTA and 5 mm GST‐Calmodulin to remove unincorporated material. The sample was diluted with 100 mL Extraction Buffer (50 mm Hepes‐KOH pH 7.5, 200 mm potassium acetate, 7 mm magnesium acetate, 2 mm DTT, 0.1 mm CaCl_2_). Samples were incubated with glutathione spin columns (Pierce) for 30 min. Flow‐through material was collected and used as precleared material for the extraction assay.

Extraction reactions were set up using precleared material with a final concentration of 3 mm Msp1 construct normalized per hexamer, 1 mg·mL^−1^ bovine serum albumin (BSA), 5 μm SGTA, and 5 μm calmodulin. Reactions were brought up to final volume with extraction buffer and were performed in triplicate. Reaction tubes were incubated at 30 °C for 1–2 min before the addition of 80 mm ATP. Reactions were then incubated at 30 °C for 25 min before the addition of 8 μm MBP‐Ubiquitin‐mNG2. They were incubated for another 5 min before transfer to TLA‐120.1 centrifuge tubes. Samples were pelleted at 100 000 ***g*** for 30 min. 20 μL of supernatant was taken from the top of the samples following centrifugation. These samples were incubated with 1.5 μm LgBiT. Samples were brought up to a total volume of 50 μL with extraction buffer. Following incubation, samples and full signal controls were transferred to individual wells of a white half‐volume 96‐well plate. 20 μL of Promega furimazine was added to each well. Luminescence was read at 470 nm with a 1 mm read height with a 1 s integration every 32 s for 10 min. Peak luminescence values were used for the calculation of percent signal.

### Analytical size exclusion chromatography

Analytical size exclusion chromatography was performed on a SEC S200 Increase 3.2/300 GL column (Cytiva) equilibrated in 20 mm Tris pH 7.5, 100 mm NaCl, 0.5 mm TCEP. A total of 0.1 mL of sample containing 1.2 mg·mL^−1^ (5 μm hexamer/pseudohexamer) Msp1, 2 mm ATP, 2 mm MgCl_2_ was loaded onto the column at a flow rate of 0.1 mL·min^−1^.

### Protease protection assay

A protease protection assay was performed on precleared material used for extraction assays. The final reaction had a total volume of 10 μL and contained 7 μL of precleared material, 2 U of thrombin protease, and 1% of Triton X‐100 (where indicated). Samples were incubated at room temperature for 1 h and then 2 μL of 100 mm PMSF was added. The samples were reverse quenched into 90 μL of boiling 1% sodium dodecyl sulfate and incubated at 95 °C for 10 min. Anti‐HiBiT western blot was performed with a 1:5000 dilution of mouse anti‐HiBiT (Promega, Clone 30E5, Madison, WI, USA) and a 1:10 000 dilution of goat anti‐mouse‐HRP secondary antibody (Invitrogen, Carlsbad, CA, USA).

### Yeast complementation assay

Strains and plasmids used in the study are described in Tables 1 and 2. For complementation assays in *S. cerevisiae*, *msp1Δ*, *get3Δ* W303‐1 yeast were generated by integrating *MSP1*::KanMX and *GET3*::NatMX using homologous recombination in WT 303–1 strain. Msp1 constructs were cloned into a *CEN* and *LEU2* vector with the flanking 281‐bp upstream and 261‐bp downstream as the respective promoter and terminator. Msp1 construct expression in *S. cerevisiae* was judged by anti‐FLAG western blot with a 1:10 000 dilution of rabbit anti‐FLAG antibody (Invitrogen) and a 1:10 000 dilution of goat anti‐rabbit‐HRP secondary antibody (Proteintech, Rosemont, IL, USA). Complementation was performed as described [26]. Briefly, haploid W303‐1 cells containing the appropriate plasmid were grown overnight in SD ‐LEU with 2% glucose. Cultures were diluted to 0.02 OD_600_ in fresh SD‐LEU media until mid‐log phase. Cultures were washed 3× with sterile water and diluted to 1 OD_600_. Samples were serially diluted 5× and then spotted onto SD‐LEU plates containing either 2% glucose or 2% glycerol and grown at 30 °C. Images are representative of N > 2 trials.

**Table 1:** Strains used in this study.

Strain	Genotype	References
CS02_DGY	*MATa ura3‐52 trp1Δ2 leu2‐3_112 his3‐11 ade2‐1 can1‐100*, *msp1::kanMX*	This study
CS013_DGY	*MATα ura3‐52 trp1Δ2 leu2‐3_112 his3‐11 ade2‐1 can1‐100*, *get3::natMX*	This study
CS015_DGY	*MATa ura3‐52 trp1Δ2 leu2‐3_112 his3‐11 ade2‐1 can1‐100,msp1::kanMX*, *get3::natMX/p34_DG*	This study
CS060_DGY	*MATa ura3‐52 trp1Δ2 leu2‐3_112 his3‐11 ade2‐1 can1‐100*, *msp1::kanMX*, *get3::natMX/p196_DG*	This study
CS061_DGY	*MATa ura3‐52 trp1Δ2 leu2‐3_112 his3‐11 ade2‐1 can1‐100*, *msp1::kanMX*, *get3::natMX/p197_DG*	This study
CS062_DGY	*MATa ura3‐52 trp1Δ2 leu2‐3_112 his3‐11 ade2‐1 can1‐100*, *msp1::kanMX*, *get3::natMX/p198_DG*	This study
CS063_DGY	*MATa ura3‐52 trp1Δ2 leu2‐3_112 his3‐11 ade2‐1 can1‐100*, *msp1::kanMX*, *get3::natMX/p199_DG*	This study
CS064_DGY	*MATa ura3‐52 trp1Δ2 leu2‐3_112 his3‐11 ade2‐1 can1‐100*, *msp1::kanMX*, *get3::natMX/p200_DG*	This study

**Table 2:** Plasmid used in this study.

Plasmid	Description	References
p34_DG	*pRS315*	
p196_DG	*CEN*, *LEU2*, P_MSP1_‐MSP1(WT)‐MSP1(WT)‐3XFLAG‐T_MSP1_	This study
p197_DG	*CEN*, *LEU2*, P_MSP1_‐MSP1‐MSP1(EQ)‐3XFLAG‐T_MSP1_	This study
p198_DG	*CEN*, *LEU2*, P_MSP1_‐MSP1(EQ)‐MSP1(WT)‐3XFLAG‐T_MSP1_	This study
p199_DG	*CEN*, *LEU2*, P_MSP1_‐MSP1(WT)‐3XFLAG‐T_MSP1_	This study
p200_DG	*CEN*, *LEU2*, P_MSP1_‐MSP1(EQ)‐3XFLAG‐T_MSP1_	This study
p202_DG	*CEN*, *HIS3*, P_TEF1_‐EGFP‐T_TEF1_	This study
p203_DG	*CEN*, *HIS3*, P_TEF1_‐mCherry‐T_TEF1_	This study
p204_DG	*CEN*, *HIS3*, P_TEF1_‐EGFP‐E2A‐mCherry‐T_TEF1_	This study
p205_DG	*CEN*, *HIS3*, P_TEF1_‐EGFP‐Pex15ΔC30‐E2A‐mCherry‐T_TEF1_	This study
p456_DG	His_10_‐bdNedd8‐His_6_‐Msp1 WT‐2xStrep Monomer	This study
pHF003	His_6_‐TEV‐Msp1 E193Q Monomer	[47]
p454_DG	His_10_‐bdNedd8‐His_6_‐WT‐WT‐2xStrep Dimer	This study
p447_DG	His_10_‐bdNedd8‐His_6_‐WT‐EQ‐2xStrep Dimer	This study
p455_DG	His_10_‐bdNedd8‐His_6_‐EQ‐WT‐2xStrep Dimer	This study
pHF027	GST‐3C‐SGTA	[47]
pHF050	GST‐3C‐Calmodulin	[47]
p098_DG	His_6_‐MBP‐Ubiquitin‐mNG2	[47]
p048_DG	His_6_‐3C‐LgBiT	[47]
pHF122	His_6_‐3C‐Sumo‐Sec22‐HiBiT	[47]

### Microscopy

Immunofluorescence microscopy was carried out as previously described [48]. Briefly, cells were fixed in 4% formaldehyde for 1 h at 30°C. Following fixation, cells were spheroplasted using zymolyase 100 T and subsequently permeabilized with methanol. Immunostaining was performed using a monoclonal anti‐FLAG antibody (F7425; Millipore Sigma, Burlington, MA, USA) along with a co‐expressed mitochondrial‐targeted RFP marker. Secondary detection was carried out using a goat anti‐mouse IgG (H + L) Alexa Fluor 488‐conjugated antibody (A‐11001; Thermo Fisher). Fluorescence imaging was conducted on a Nikon TiE2 inverted microscope equipped with an Apo100× objective (NA 1.45) and captured with an Orca Flash 4.0 cMOS (Hammamatsu, Bridgewater, NJ) camera and NIS‐Elements software (Nikon). Image acquisition was controlled using the NIS‐Elements software (Nikon), with identical acquisition settings maintained across all samples in an experiment. Images were uniformly cropped and adjusted using Adobe Photoshop (Adobe Systems Inc., San Jose, CA, USA).

### Flow cytometry assay

GFP‐Pex15ΔC30‐E2A‐mCherry was cloned into a *CEN* and *HIS3* based vector and transformed into *msp1Δ*, *get3Δ* W303‐1 yeast containing an Msp1 construct on a *CEN‐* and LEU2‐based vector. Cells were grown in SD‐HIS‐LEU media at 30 °C until they reached OD_600_ ~ 3.0, then diluted to OD_600_ = 1 in sterile water media. Flow cytometry was completed on a BD Biosciences LSR15 cytometer and further analysis was completed using FlowJo as described previously [49].

## Results

### Development of covalently linked dimers

A major obstacle in the development of linked dimers is the design of a linker that will not significantly impact the function of the enzyme. The genetically encoded linker needs to have sufficient length and flexibility to minimize disruption of catalytic activity. Based on the cryo‐EM structure of soluble Msp1 (PDB = 6PDW), the distance between N and C‐termini in adjacent subunits ranges from 55 to 76 Å [16]. In principle, the shortest distance could be bridged by 16 fully‐extended amino acids, whereas the longest distance would require more than 22 amino acids. These are only rough estimates, as the termini are not visible in the structure, and any linker between subunits would need to adopt a longer, more flexible conformation to avoid steric clashes. To determine the optimal linker length, we generated three different soluble constructs consisting of two wild‐type subunits with linkers of 14, 25, or 35 amino acids.

We measured ATPase rates at a single concentration of ATP as a proxy for how the linker lengths affect Msp1 activity. The construct with a 35‐residue linker had the smallest decrease in ATPase activity compared to the WT monomer (Fig. 1B). The 35‐residue linker is attractive because it can accommodate even the longest possible distance between subunits; however, it also carries the risk of allowing alternative arrangements of subunits. While we cannot definitively rule out this risk, AlphaFold3 modeling of the hexamer shows that the top five models all have the expected arrangements of alternating subunits (Fig. 1C).

The final version of the linked Msp1 constructs has a 35‐residue linker and was generated by gene synthesis to allow for each subunit to have identical protein sequences but unique DNA sequences. The linker is composed of small, flexible, hydrophilic amino acids and contains a unique restriction enzyme site, allowing for individual subunits to be replaced with mutated versions via restriction cloning. Given the large number of potential permutations, the linker sequence itself was not systematically optimized beyond initial screening of linker length.

We generated two versions of the linked dimers, a soluble version for *in vitro* assays and a full‐length version for *in vivo* assays (Fig. 1D). Soluble constructs have a deletion of the first 32 amino acids in each subunit. There is an N‐terminal His_6_ tag to allow for anchoring to the liposomes in the *in vitro* extraction assay. There is also a C‐terminal twin‐strep tag to allow for a selective purification of full‐length constructs from truncation products. To rule out any potential effects of the C‐terminal strep tag, we also generated an Msp1 monomer with a C‐terminal twin strep tag and purified this construct in the same manner as the linked dimers. We hereafter refer to this construct as the WT monomer. For *in vivo* assays, we generated “full‐length” constructs where the first subunit contains the native TMD and the second subunit has a deletion of the first 32 amino acids to remove the TMD (Fig. 1D). To allow detection by western blot, we also added a 3× FLAG tag at the C‐terminus.

### The E193Q mutation does not act as a dominant negative for ATP hydrolysis

**Fig. 2:**
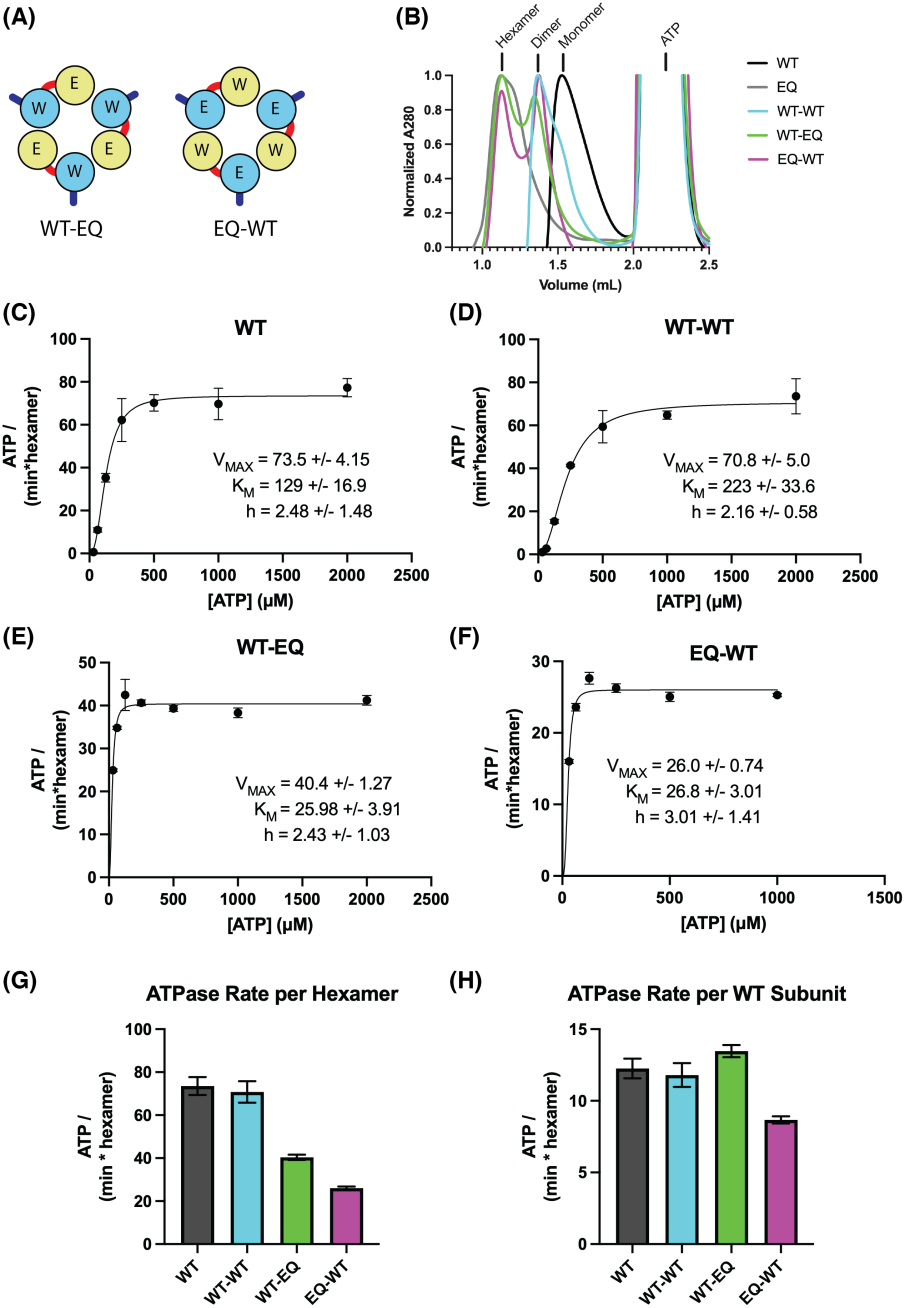
The E193Q mutation does not act as a dominant negative. (A) Diagram showing the organization of WT‐EQ and EQ‐WT linked dimers. The N‐terminal membrane anchor is dark blue and the covalent linker between subunits is red. (B) Size exclusion chromatography on an S200 Increase 3.2/300 column shows that WT‐EQ and EQ‐WT linked dimers exhibit properties of both the WT and EQ subunits. Michaelis–Menten curve for ATP hydrolysis for the (C) WT monomer, (D) WT‐WT dimer, (E) WT‐EQ dimer, and (F) EQ‐WT dimer. Error bars show standard deviation from three replicates. The data was fit to the Hill form of the Michaelis–Menten equation, *V* = *V*_MAX_/(1 + (*K*_M_/[S])^h^). *V*_Max_, *K*_M_, and the Hill coefficient (h) are listed with the 95% confidence interval. (G) *V*_Max_ for ATPase activity calculated as ATP per minute per hexamer. Error bars show the 95% confidence interval. (H) As in G, but *V*_Max_ for ATPase activity is calculated at ATP per minute per WT subunit. Note that WT monomer, WT‐WT, and WT‐EQ all have similar ATPase rates per WT subunit. Error bars show the 95% confidence interval.

To better understand how Msp1 couples ATP hydrolysis to mechanical work, we generated three constructs containing a mixture of wild‐type (WT) and E193Q (EQ) Walker B mutations. The Walker B mutation allows ATP to bind to a subunit but prevents hydrolysis. We refer to these constructs as WT‐WT, WT‐EQ, and EQ‐WT (Fig. 2A).

As an initial characterization of the linked dimers, we performed size exclusion chromatography on the soluble constructs. Previous work showed that WT Msp1 runs predominantly as a monomer, whereas the ATPase‐deficient E193Q mutant runs as a hexamer [26]. As expected, the WT‐WT dimer elutes between the WT monomer and E193Q hexamer peaks (Fig. 2B). Interestingly, the WT‐EQ and EQ‐WT constructs show two peaks. One peak overlaps with the E193Q hexamer, and the other overlaps with the WT‐WT dimer, indicating that both the WT and EQ subunits influence construct oligomerization. Importantly, none of the constructs eluted earlier than the E193Q hexamer, suggesting that the linked constructs assemble into pseudohexameric trimers of dimers rather than larger oligomeric species.

To assess enzymatic activity, we measured ATP hydrolysis. Importantly, the WT‐WT dimer had a nearly identical *V*_Max_ as the WT monomer, and the *K*_M_ was within twofold, indicating that the linker had minimal effect on ATP hydrolysis (Fig. 2C,D). Interestingly, the *K*_M_ for both the WT‐EQ and EQ‐WT constructs was ~10‐fold lower than the WT‐WT dimer, potentially due to allosteric effects from the neighboring EQ subunit. Indeed, the kinetic data was best fit by the Hill form of the Michaelis‐Menten equation, with all four constructs exhibiting a Hill coefficient between 2 and 3, suggesting that there is significant allosteric communication between subunits.

As expected for constructs with only 50% active subunits, the *V*_Max_ for both WT‐EQ and EQ‐WT constructs was lower than the WT monomer (Fig. 2E,F). The *V*_Max_ for the EQ‐WT construct was ~65% of the WT‐EQ construct, suggesting the effect of the EQ subunit may be position specific. However, all constructs retained ATPase activity, indicating that the EQ subunit does not act in a dominant negative fashion (Fig. 2G). Furthermore, when the ATPase rate is normalized to the number of WT subunits, we observed no difference in the rate of ATP hydrolysis between WT‐WT and WT‐EQ (Fig. 2H). This indicates that the neighboring EQ subunit in this construct does not significantly inhibit ATPase activity in the WT subunit. We conclude that the linked constructs assemble into enzymatically active pseudohexamers and that subunits with the E193Q mutation do not act in a dominant negative manner for ATP hydrolysis.

### Linked constructs retain activity *in vivo*

**Fig. 3:**
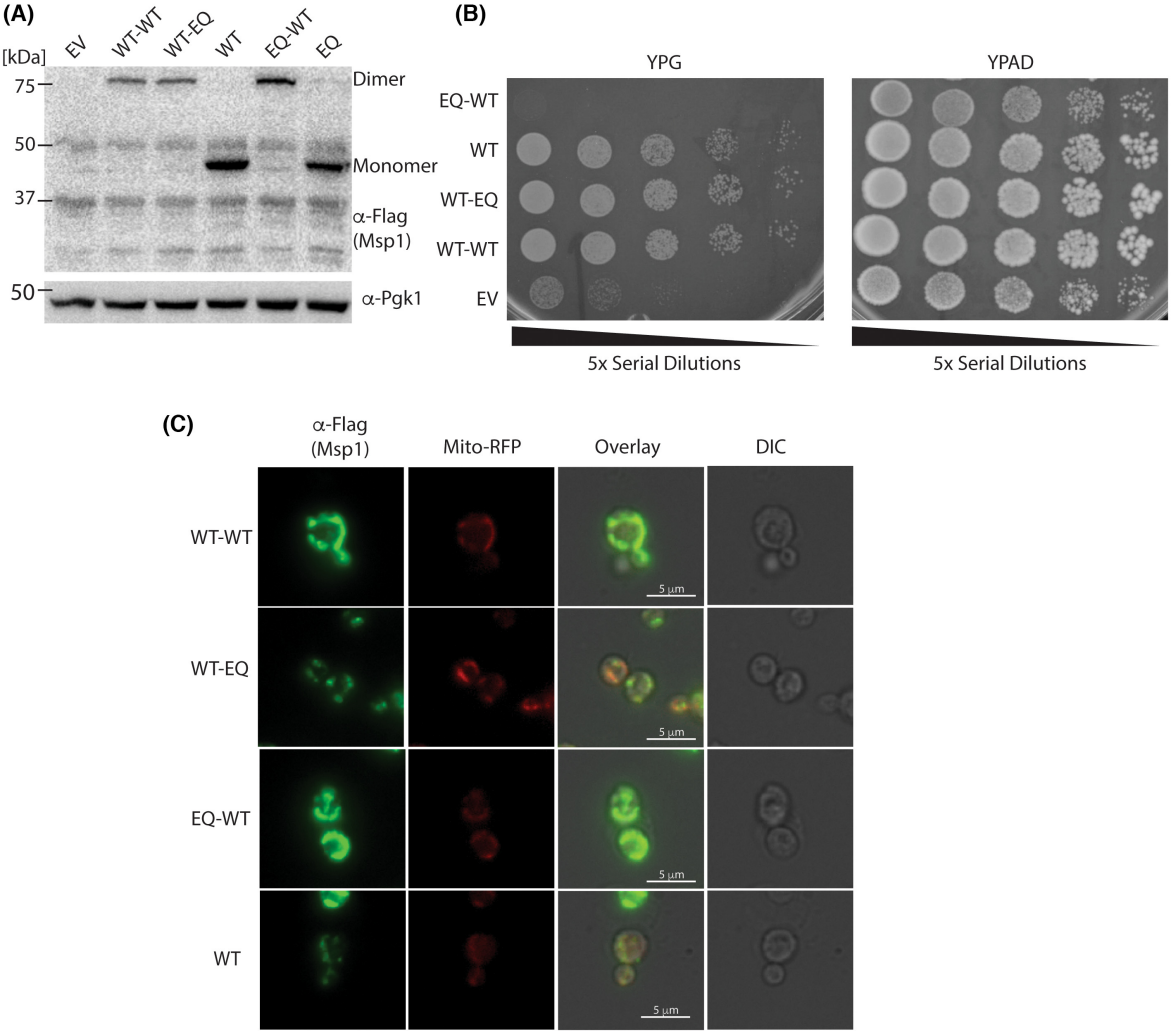
Linked constructs retain activity *in vivo*. (A) Anti‐FLAG western blot shows approximately equal expression of linked dimers. Representative blot from two biological replicates. (B) Complementation assay shows that WT‐WT and WT‐EQ can rescue growth of *msp1Δ*, *get3Δ* cells on glycerol, whereas EQ‐WT cannot. Representative image from two biological replicates. (C) Microscopy shows that linked dimers localize to the mitochondria. Scale bar is 5 μm.

To test whether the linked dimers are active *in vivo*, we cloned the full‐length version into a centromeric plasmid with the native Msp1 promoter. Anti‐FLAG western blots show relatively equal expression across the three dimers (Fig. 3A). The dimer bands have lower signal intensity than monomeric Msp1. However, only three copies of the dimers are required to form a functional hexamer rather than six copies of the monomer. Thus, the overall concentration of hexamer is roughly comparable between the monomeric and dimeric constructs.

As a first test of Msp1 activity *in vivo*, we performed a complementation assay. Simultaneous deletion of *MSP1* and *GET3* leads to a loss of oxidative phosphorylation, which can be assayed by growth on the nonfermentable carbon source glycerol [26]. We therefore generated *msp1Δ*, *get3Δ* yeast strains complemented with a centromeric plasmid. As expected, complementation with the empty vector failed to rescue growth on glycerol, whereas plasmid‐based expression of WT Msp1 provided a complete rescue. Surprisingly, complementation with both WT‐WT and WT‐EQ provided a complete rescue, indicating that the EQ subunits in this construct do not act as a dominant negative.

The EQ‐WT construct provided no rescue and appeared to have a slight toxic effect compared to the empty vector control (Fig. 3B). To rule out that the observed activity differences arise from differences in mitochondrial localization, we performed immunofluorescent microscopy analysis. We observed that all constructs localize to the mitochondria as expected (Fig. 3C). There were some differences in the distribution of the constructs on the mitochondria, but this did not appear to correlate with activity levels as the WT‐WT and EQ‐WT displayed similar distributions on the mitochondria despite the former retaining activity and not the latter. We conclude that the WT‐WT and WT‐EQ constructs retain functionality *in vivo* while the EQ‐WT construct is inactive.

### Linked dimers drive extraction of Pex15ΔC30 *in vivo*

**Fig. 4:**
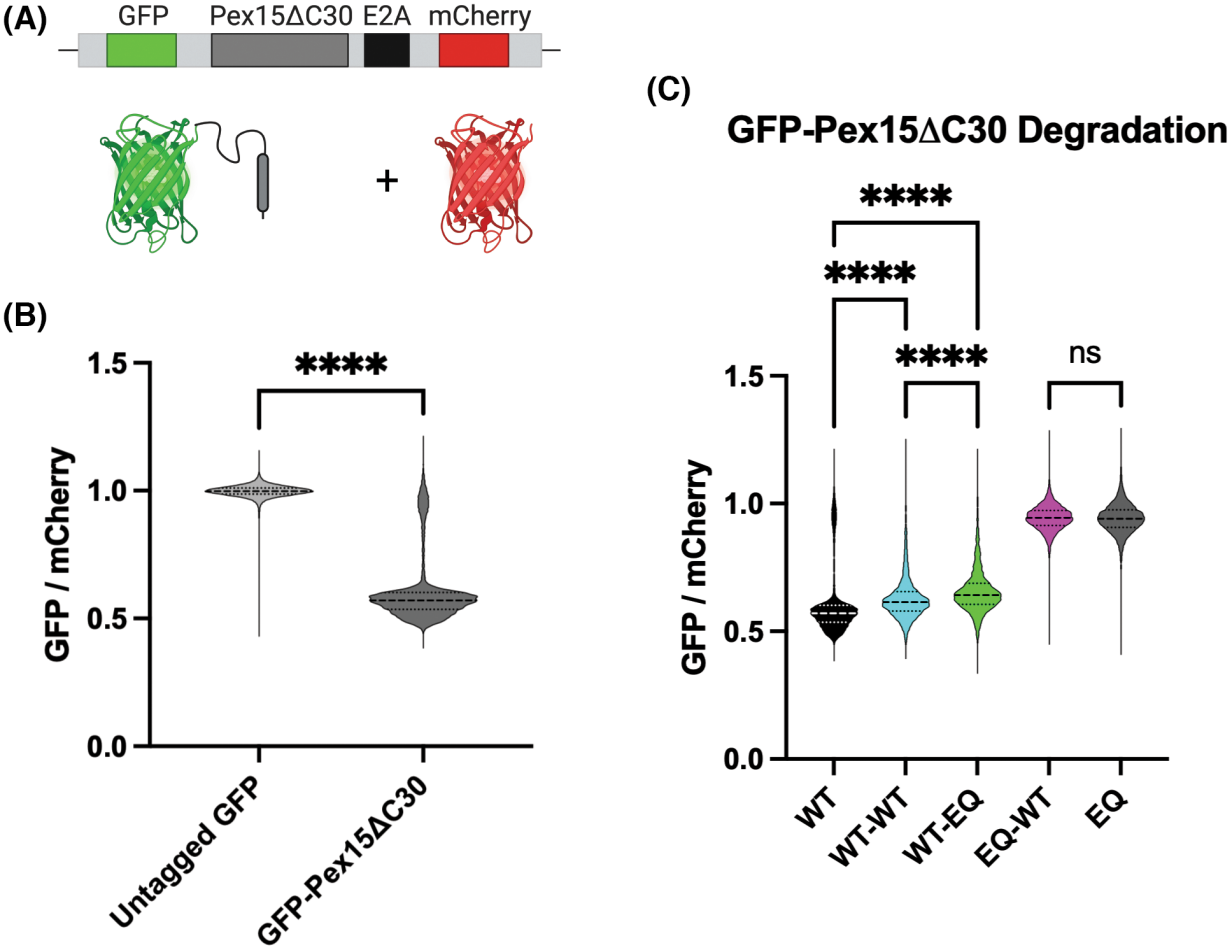
*In vivo* extraction of Pex15ΔC30. (A) Diagram of the reporter used for the *in vivo* extraction assay. (B) Flow cytometry measurement of GFP and mCherry fluorescence intensity show that appending Pex15ΔC30 onto the C‐terminus of GFP leads to degradation. *msp1Δ*, *get3Δ* yeast cells were complemented with a centromeric plasmid expressing WT Msp1. *P*‐value was calculated by an unpaired, 2‐tailed *t*‐test; ****P < 0.0001. (C) Flow cytometry measurement of GFP and mCherry fluorescence intensity show modest but significant differences in extraction activity of WT, WT‐WT, and WT‐EQ constructs. Despite retaining ATPase activity, the extraction activity of the EQ‐WT construct is not significantly different from the EQ construct. *msp1Δ*, *get3Δ* yeast cells were complemented with a centromeric plasmid expressing the indicated linked dimers. The data is representative of two biological replicates with a minimum of 5000 cells per replicate. *P*‐values were calculated by 1‐way ANOVA with Dunnett's *post hoc* test; ****P < 0.0001; ns, not significant.

To get a higher resolution view of Msp1 extraction activity *in vivo*, we used a GFP/mCherry reporter assay [49]. Briefly, GFP and mCherry are expressed on a polycistronic vector separated by the codon skipping E2A site, giving rise to equal expression of GFP and mCherry in the cell (Fig. 4A). We then fused the known Msp1 substrate Pex15ΔC30 to the C terminus of GFP. The ΔC30 truncation causes Pex15 to constitutively mislocalize to the mitochondria [27], where it is extracted by Msp1 and eventually degraded [30]. Msp1 activity is measured by the ratio of GFP to mCherry in cells via flow cytometry, with a lower GFP/mCherry ratio indicating higher levels of Msp1 activity. Control reactions with *msp1Δ*, *get3Δ* yeast complemented with WT Msp1 on a centromeric plasmid show that the GFP/mCherry ratio is ~1 with untagged GFP and decreases to ~0.5 when Pex15ΔC30 is fused to GFP (Fig. 4B).

We then used this assay to test the activity of the linked dimers (Fig. 4C). Consistent with the complementation assay, the EQ‐WT construct was indistinguishable from the negative control EQ monomer. The WT monomer, WT‐WT, and WT‐EQ constructs showed robust activity. We observed a modest but significant difference between the three active constructs. The WT monomer was the most active, WT‐EQ was the least active, and WT‐WT was intermediate between the two.

### *In vitro* substrate extraction requires highly fluid membranes

To further probe the extraction activity of the linked constructs, we turned to our *in vitro* extraction assay, which provides full experimental control over both the substrate and lipid environment. This assay utilizes a split‐luciferase system, with substrate extraction monitored by luminescence [47]. To rule out potential differences in substrate extraction due to the number of TMDs in the liposomes, we performed the extraction assay with soluble Msp1 as previously described [34]. These constructs are anchored to the liposomes via the N‐terminal His_6_ tag, which interacts with the DGS‐Ni‐NTA lipid that makes up 2% of the mole fraction of the liposomes.

We previously demonstrated that extraction of the TMD from the lipid bilayer presents the largest thermodynamic barrier for substrate extraction and that Msp1 extraction activity is highest in more fluid lipid bilayers [47]. We therefore performed the extraction assay with our standard Sec22 model substrate reconstituted into liposomes made up of 98% phosphatidyl choline with either 18:1, 18:1 *trans*, or 18:2, 18:2 *cis* acyl chains, hereafter referred to as 18:1 and 18:2 liposomes, respectively.

**Fig. 5:**
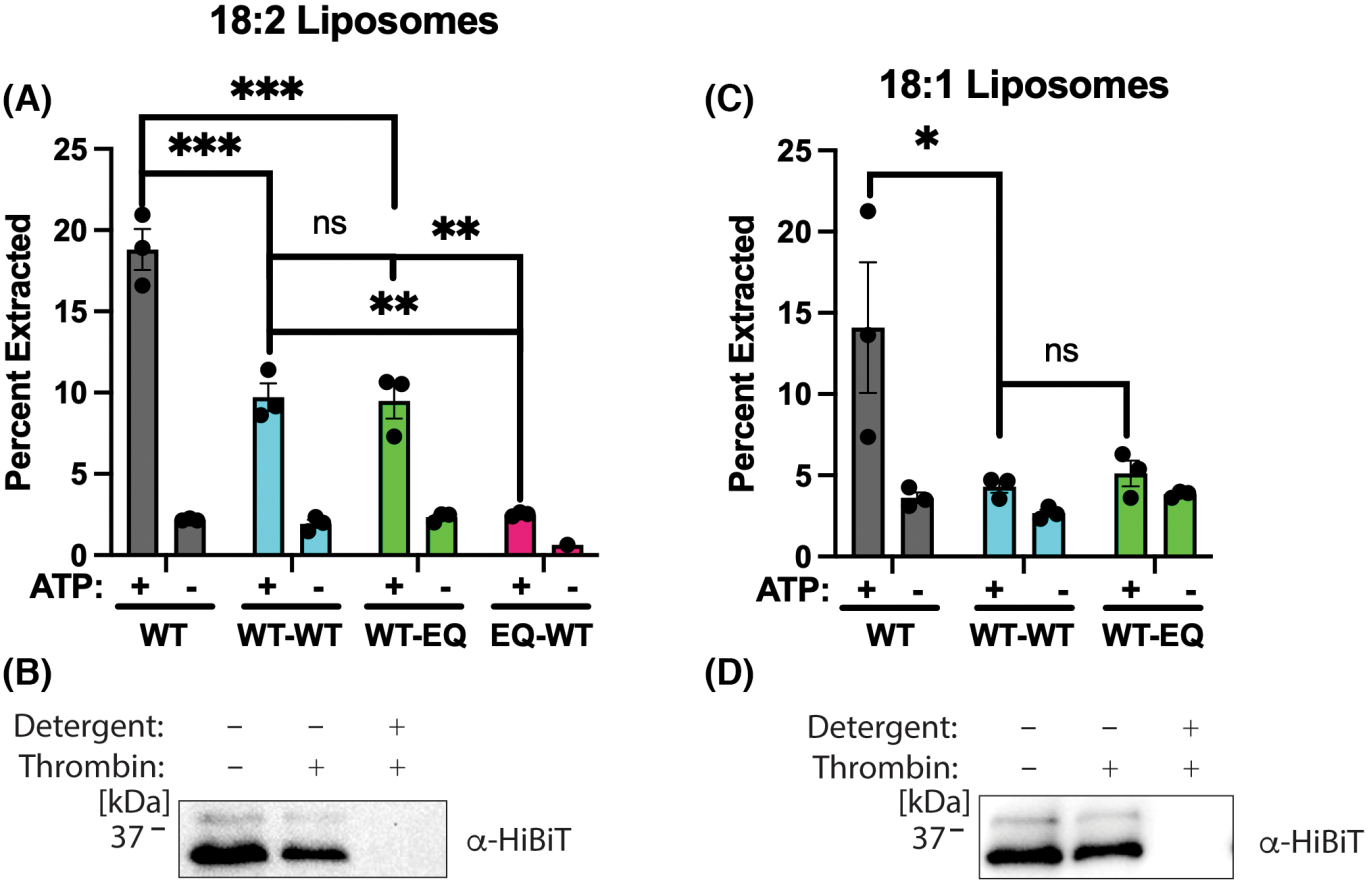
*In vitro* substrate extraction requires highly fluid membranes. (A) The WT‐WT and WT‐EQ constructs show ATP‐dependent substrate extraction in 18:2 liposomes. Error bars show standard error of the mean for three replicates. *P*‐values were calculated by 1‐way ANOVA with Dunnett's *post hoc* test; ***P < 0.001, **P < 0.01, ns, not significant. (B) Protease protection assay shows that the model substrate is properly oriented in 18:2 liposomes. Anti‐HiBiT western blot of substrate in precleared 18:2 liposomes shows minimal loss of signal upon addition of thrombin protease. Addition of detergent solubilizes the liposomes, allowing the protease to access the thrombin protease site. The resulting HiBiT peptide is not resolvable by SDS PAGE/western blot. (C) The WT‐WT and WT‐EQ constructs show no ATP‐dependent substrate extraction in 18:1 liposomes. Error bars show standard error of the mean for 3 replicates. *P*‐values were calculated by 1‐way ANOVA with Dunnett's *post hoc* test; *P < 0.05, ns, not significant. (D) Protease protection assay shows that the substrate is properly oriented in 18:1 liposomes. Similar to B, anti‐HiBiT western blot of substrate in precleared 18:1 liposomes.

As expected, extraction activity was higher in the more fluid 18:2 liposomes than in the 18:1 liposomes [47] (Fig. 5A,C). A protease protection assay shows that the substrate is properly oriented, with the C‐terminal HiBiT tag in the lumen of the liposomes [47] (Fig. 5B,D). The model substrate contains a C‐terminal thrombin protease site between the TMD and HiBiT tag.

The HiBiT tag only becomes protease accessible upon addition of detergent, which solubilizes the liposomes. Similar to the *in vivo* extraction assay, the WT monomer had the highest level of extraction, indicating the linker is inhibiting extraction activity despite no difference in the *V*_Max_ for ATP hydrolysis. Interestingly, the WT‐WT and WT‐EQ constructs were only active in the more fluid 18:2 liposomes. Despite the difference in ATPase rate, we observed minimal differences in extraction between the WT‐WT and the WT‐EQ constructs. Similar to the *in vivo* data, the EQ‐WT construct showed negligible extraction even in the highly fluid 18:2 liposomes. We conclude that the linked dimers are active for substrate extraction *in vitro*, that extraction activity depends on the fluidity of the lipid bilayer, and that the linker reduces substrate extraction *in vitro*.

## Discussion

AAA+ proteins are ubiquitous molecular motors that are involved in numerous essential cellular processes. These hexameric proteins convert the energy from ATP hydrolysis into mechanical work, resulting in substrate remodeling. While significant effort has been devoted to understanding how AAA+ proteins drive protein unfolding, comparatively little is known about how AAA+ proteins extract membrane proteins from the lipid bilayer. Here, we used the mitochondrial AAA+ protein Msp1 as a model system to examine this important topic. We generated covalently linked Msp1 dimers containing a mixture of wild‐type and ATPase deficient E193Q Walker B mutations. The resulting trimers of dimers have varying rates of ATP hydrolysis that we used to probe how ATP hydrolysis is coordinated between Msp1 subunits for TMH extraction from the lipid bilayer.

While it is formally possible that the long linker allows for alternative arrangements of subunits, multiple lines of evidence argue that the dimers assemble into functional pseudohexamers as expected. First, AlphaFold3 modeling shows the expected arrangement of subunits in the proper orientation. Second, size exclusion chromatography shows the expected distributions of dimers and hexamers with no formation of larger oligomeric species. Third, the WT‐WT construct has the same *V*_Max_ for ATP hydrolysis as the WT monomer, indicating that the linker has minimal effect on ATP hydrolysis. Finally, the WT‐EQ and EQ‐WT constructs exhibit the expected properties of constructs with a mixture of WT and EQ subunits. They run as a mixture of dimers and hexamers on size exclusion, and the *K*_M_ for ATP hydrolysis is significantly tighter than for the WT‐WT construct, both of which could be due to allosteric effects of the EQ subunit.

Surprisingly, the EQ‐WT construct was active for ATP hydrolysis but showed no extraction activity under any conditions tested. While this construct had slightly lower rates of ATP hydrolysis compared to the WT‐EQ construct, the complete lack of activity suggests that having an ATPase‐inactive subunit next to the membrane anchor allows it to act in a dominant negative fashion for substrate extraction, but not for ATP hydrolysis.

**Fig. 6:**
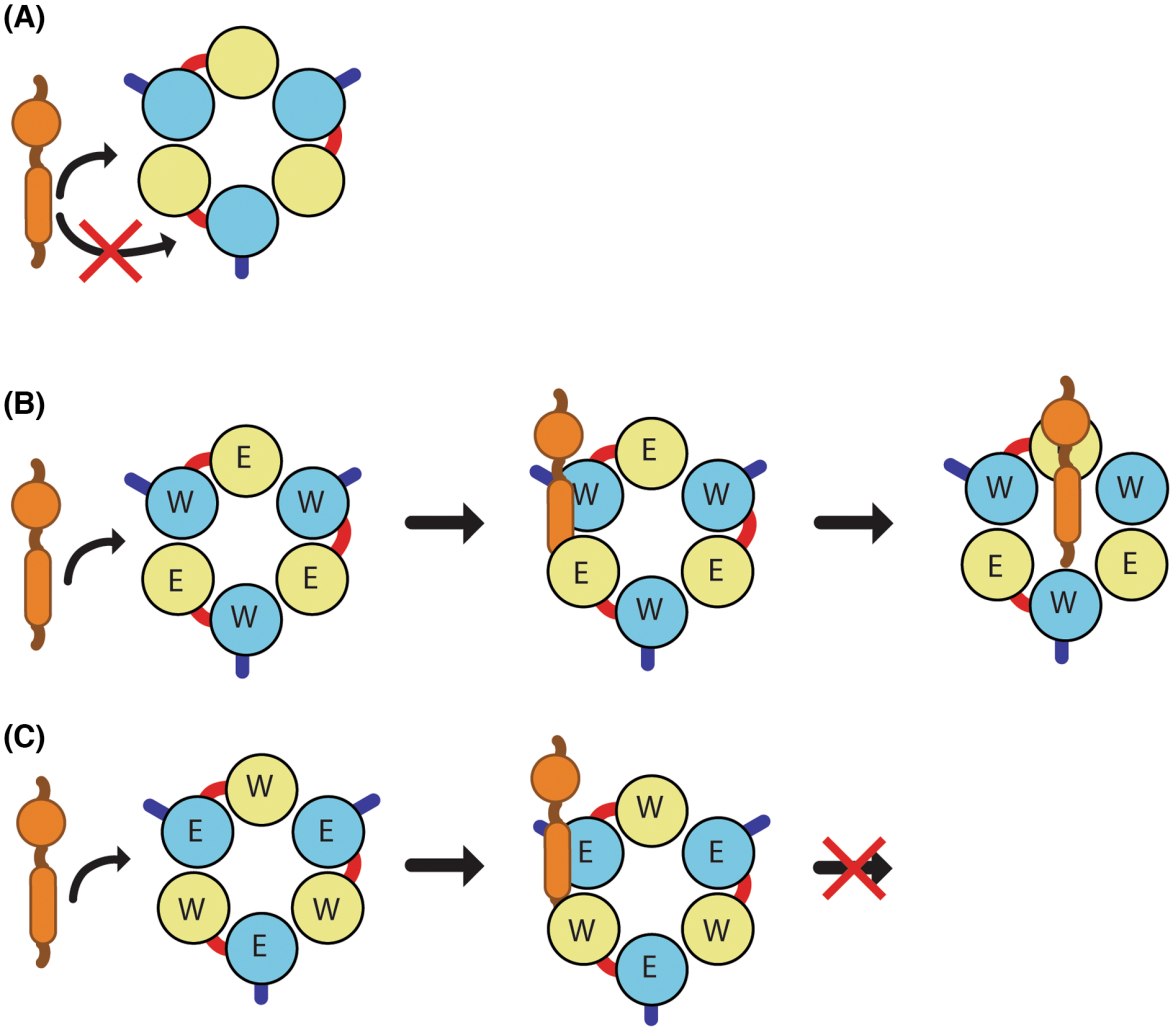
Proposed model for how linkers and subunit arrangement can inhibit substrate extraction without affecting ATPase activity. (A) To gain access to the central pore and be extracted, the substrate laterally diffuses through the seam in the hexamer. The linker restricts the sites available for lateral diffusion. (B) For the WT‐EQ construct, the substrate will first engage with a WT subunit and then will continue to laterally diffuse into the pore for extraction. (C) For the EQ‐WT construct, the substrate will first engage with an EQ subunit, which acts as a substrate trap and inhibits substrate extraction by preventing lateral diffusion into the pore.

A possible hint into the location‐specific effects of the EQ subunit comes from comparing the extraction activity of the WT‐WT dimer and WT monomer constructs. These constructs have the same *V*_Max_ for ATP hydrolysis, but the WT monomer is more active for substrate extraction *in vitro* and *in vivo*, suggesting that the linker has a negative effect on substrate extraction. We and others have suggested that substrates may access the central pore of Msp1 via lateral diffusion through the seam in the lock washer observed in multiple cryo‐EM structures [16, 24, 47]. This model is particularly enticing as the seam subunit uniquely exposes a hydrophobic patch within the N‐domain that binds to exposed hydrophobic residues in the substrate [50]. The linker in our dimeric constructs will restrict lateral diffusion such that only the first subunit in the dimer can form a seam, thereby explaining why the WT‐WT construct has lower extraction activity than the WT monomer despite no difference in ATPase rates (Fig. 6A). An extension of this model is that the substrate will first engage with a WT subunit in the WT‐EQ construct and an EQ subunit in the EQ‐WT construct (Fig. 6B,C). As the E193Q mutation is known to act as a substrate trap, this could potentially explain the differences in substrate extraction for the WT‐EQ and EQ‐WT constructs. Rigorously testing this model should be a priority.

Another interesting observation from our data is that the WT‐WT and WT‐EQ constructs had similar levels of substrate extraction *in vitro* despite a twofold difference in *V*_Max_ for ATP hydrolysis. We previously demonstrated that the largest thermodynamic barrier for substrate extraction is removal of the TMH from the lipid bilayer [47]. Consistent with this data, both constructs were equally active in the highly fluid 18:2 liposomes but were essentially inactive in the more rigid 18:1 liposomes. Interestingly, the OMM is one of the most fluid membranes in the cell, whereas the peroxisome, where Msp1 is also localized, is much less fluid [51]. One possible interpretation is that for Msp1 to be functional in the peroxisome, it must have a higher rate of ATP hydrolysis than is required for extraction of typical single‐pass substrates from the OMM; similar to how a car does not utilize all available horsepower when cruising on the highway. We propose that the “reserve horsepower” required for Msp1 to function in the peroxisome may allow it to extract particularly challenging substrates from the OMM. Testing this model will be another important area of future investigation.

The efficiency of substrate processing depends on the coordination of ATP hydrolysis between subunits in the Msp1 homohexamer. Recent cryo‐EM structures of *Chaetomium thermophilum* Msp1 and human ATAD1 show a canonical right‐handed spiral arrangement of subunits that has been observed in many other AAA+ proteins and generally supports the SC/2R mechanism of ATP hydrolysis [15, 16]. Our data strongly suggest that Msp1 does not operate strictly through the SC/2R mechanism. Indeed, there is no difference in the rate of ATP hydrolysis between the WT‐WT and WT‐EQ constructs when normalized to the number of WT subunits. Furthermore, the WT‐EQ and WT‐WT constructs show essentially equal levels of activity in multiple different extraction assays. While further work is needed to determine the details for how Msp1 subunits coordinate ATP hydrolysis, we conclude that Msp1 does not operate solely by the SC/2R mechanism of ATP hydrolysis but instead shows mechanistic plasticity. We propose that this mechanistic plasticity may provide robustness to prevent stalling during the translocation of challenging substrates, similar to mechanisms used by soluble AAA+ proteins [52, 53, 54, 55, 56].

The dissonance between models of ATP hydrolysis from structural and biochemical experiments appears to be a recurring theme in the study of AAA+ proteins and may hint at deeper mechanisms [57]. While cryo‐EM structures of diverse AAA+ proteins almost universally support the SC/2R mechanism, biochemical experiments suggest that there is likely more mechanistic plasticity than is revealed by these static structures [12, 55, 58]. For example, single turnover experiments with the AAA+ protein ClpA showed a step size of 14 amino acids alone or 5 amino acids when in complex with the ClpP Protease [59, 60]. Likewise, covalently linked ClpX hexamers containing multiple ATPase inactive subunits are still capable of unfolding and translocating substrates [20]. Single molecule optical trapping experiments with ClpXP showed a basic step size of 5–8 to 10–13 amino acids [41]. It is possible that the larger step sizes are actually composed of multiple unresolved SC/2R steps, but the differing kinetics of substrate translocation and ATP hydrolysis argue against this possibility [19]. The inconsistency between biochemical and structural data has given rise to other mechanisms for coordinating ATP hydrolysis between subunits, such as the Probabilistic Anti‐clockwise Long‐Step (PA‐LS) or Sequential Clockwise/6‐residue (SC/6R) [12, 19].

Overall, our work sheds light on how Msp1 utilizes ATP hydrolysis to perform mechanical work both *in vitro* and *in vivo*. It also raises interesting questions regarding the minimum rates of ATP hydrolysis required to overcome the thermodynamic barrier of TMH extraction from a lipid bilayer, how Msp1 coordinates ATP hydrolysis between subunits, and how substrates access the axial pore. The Msp1 dimers presented here will be a powerful tool for addressing these important questions.

## Author contributions

Conceptualization: DG, BA, BAS, NW, IW, MLW; Data curation: DG, BA, BAS, NW, IW, MLW; Formal analysis: DG, BA, BAS, NW, IW, MLW; Funding acquisition: MLW; Investigation: DG, BA, BAS, NW, IW, MLW; Methodology: DG, BA, BAS, NW, IW, MLW; Project administration: MLW; Resources: DG, BA, BAS, NW, IW, MLW; Software: Not applicable; Supervision: MLW; Validation: DG, BA, BAS, NW, IW, MLW; Visualization: DG, BA, BAS, NW, IW, MLW; Writing—original draft: BAS, MLW; Writing—review and editing: DG, BA, BAS, NW, IW, MLW.

## Data Availability

The data that support the findings of this study are openly available in Mendeley Data at Mendeley Data, reference number 10.17632/8kgsvnfz2m.1.
